# Identification of specific recommendations for prehospital stroke care associated with shorter door-to-CT times – An analysis of Get with the Guidelines-Stroke registry and prehospital data

**DOI:** 10.3389/fstro.2024.1355889

**Published:** 2024-05-30

**Authors:** Layne Dylla, Hannah M. Higgins, Courtney D. Wham, Michelle Leppert, Brandy C. Ravare, Kerri A. Jeppson, Heather T. Bina, Andrew A. Monte, Sharon N. Poisson

**Affiliations:** ^1^Department of Emergency Medicine, University of Colorado School of Medicine, Aurora, CO, United States; ^2^Department of Neurology, University of Colorado School of Medicine, Aurora, CO, United States

**Keywords:** stroke, prehospital emergency care, EMS, cerebrovascular accident, ischemic stroke

## Abstract

**Objective:**

Full compliance with American Heart Association (AHA) recommendations for prehospital care of stroke patients remains low. This study aims to identify components of prehospital care associated with shorter door-to-computed tomography (CT) times.

**Methods:**

Data from a comprehensive stroke center's Get with the Guidelines-Stroke registry were supplemented by prehospital medical records for ischemic stroke patients between January 1, 2018, and December 31, 2020. Descriptive statistics and multivariable linear regression modeling was used to evaluate door-to-CT times for encounters compliant with AHA recommendations.

**Results:**

There were 621 ischemic stroke patients who presented via a prehospital provider, 452 of whom presented from the scene. Without adjusting for potential confounders, shorter door-to-CT times were observed with emergency medical services' documentation of a last-known well time, measurement of a blood glucose level, prenotification of a suspected stroke, or full compliance with AHA recommendations. Documentation of classic stroke signs, but not of a prehospital stroke scale, was also observed to have shorter door-to-CT times compared to encounters in which this did not occur.

**Conclusion:**

During prehospital care of stroke, documentation of classic symptoms, obtaining a last-known well time or time of symptom onset, obtaining a blood glucose level, prenotifying the receiving hospital of suspected stroke, and complying fully with guidelines are associated with shorter door-to-CT times. Further studies are needed to understand if a shift in prehospital provider education, focusing on these key components of care, could lead to earlier diagnosis and treatment of acute stroke.

## Introduction

In acute ischemic stroke, time is brain. Every minute a stroke goes untreated, up to 1.9 million neurons and 14 billion synapses are lost (Saver, 2006). Early recognition and treatment of stroke are key to improved outcomes. With the majority of patients with acute strokes presenting to a hospital via a prehospital provider, relying on an established stroke screening scale has been shown to increase the early recognition of stroke, allowing for early notification and preparation of the in-hospital emergency department (ED) and stroke provider teams (Fassbender et al., 2013; Oostema et al., 2015). The diagnostic sensitivity and specificity of prehospital stroke screening tools approach 80%−88% sensitivity and 90%−95% specificity depending on the precise tool used (Zhelev et al., 2019). Early prehospital recognition of stroke is associated with improved in-hospital quality of care, expeditious treatment, and improved 3-month mortality (Magnusson et al., 2022). Yet, prehospital providers have also noted that knowledge gaps regarding stroke treatments, the diversity of clinical presentation, and a lack of feedback regarding prehospital care contribute to failing to identify a stroke and lower compliance with existing guidelines (Oostema et al., 2019).

The current American Heart Association (AHA) recommendations for prehospital care of suspected stroke patients include documenting a recognized stroke screening scale, pre-notifying receiving hospitals when a stroke scale is positive, obtaining a blood glucose measurement, obtaining a 12-lead electrocardiogram (ECG), providing supplemental oxygen for hypoxia with pulse oximetry < 94%, and documenting the last-known well (LKW) time (Jauch et al., 2013; Glober et al., 2016; Powers et al., 2019) (Box 1). Additionally, the AHA provides recommendations on transport times to minimize delays in presentation to the receiving hospital (i.e., < 2 min from dispatch to being en route and < 15 min spent on scene; Jauch et al., 2013; Glober et al., 2016; Powers et al., 2019). Despite recognition of the important role of prehospital providers, full compliance with these AHA guidelines for prehospital care of suspected stroke patients is < 1% nationwide (Dylla et al., 2022). It is unknown if every guideline is essential, or which guidelines are key to improving in-hospital quality of care and patient outcomes. The primary objective of this study is to determine the association between individual recommendations for prehospital care and in-hospital quality of care for acute ischemic stroke.

Box 1 **American Heart Association recommendations for prehospital care of suspected stroke patients.**

Documentation of recognized stroke screening scale

Prenotification of receiving hospital when a stroke scale is positive

Obtain a blood glucose measurement

Obtain 12-lead electrocardiogram

Provide supplemental oxygen for hypoxia (pulse oximetry < 94%)

Document last known well time

Spend <2 min from dispatch to being en route

Spend <15 min on scene prior to transport to receiving hospital

## Methods

### Study design and population

This is a retrospective cohort study of adult patients (age 18 years or older) who presented to a single comprehensive stroke center via emergency medical services (EMS). The hospital serves as the primary receiving center for most of the eastern corridor of the state with two large EMS agencies and more than 10 smaller EMS agencies using this facility as their primary field destination for stroke. In addition, this hospital receives interhospital transfers from surrounding smaller primary stroke centers and hospitals within the broader university health system. Interfacility transfers are done through the 911 system or a transfer center where providers can specify requirements and level of care [Basic Life Support (BLS), Advanced Life Support (ALS), or critical care]. Data for all patients transported via EMS were collected, with the primary analysis focused on only those directly transported from the scene. Individual EMS agencies are subject to the protocols set forth by their medical directors. Most medical directors support protocols set forth by the regional EMS medical directors' group. In cases in which EMS suspect an acute stroke (positive stroke screening scale and LKW time of < 24 h), they are encouraged to notify the receiving hospital. A prehospital stroke alert can be called based on this information. The patient will be preregistered, and EMS will transport the patient directly to CT. A multidisciplinary team composed of a vascular neurologist, an ED physician, and an ED pharmacist will meet the patient and EMS at CT for an evaluation and discussion regarding further diagnostic evaluations and acute interventions. Any patient presenting to this comprehensive stroke center with a diagnosis of acute or subacute ischemic stroke is captured by the local Get with the Guidelines-Stroke (GWTG-S) registry and was eligible for inclusion if they had an associated prehospital run sheet available for review in the electronic medical record. Patients were admitted to the hospital between January 1, 2018, and December 31, 2020. The study was conducted under the general principles of a high-quality chart review and the STrengthening the Reporting of Observational Studies (STROBE) guidelines (Gilbert et al., 1996; von Elm et al., 2007). Given the retrospective nature, this study was conducted under a waiver of consent. It was approved by the Colorado Multiple Institutional Review Board (COMIRB #20-1369) and adhered to the principles of the Declaration of Helsinki.

### Data collection

Patient demographics, transport to the ED or direct admission, initial neurological assessment, and door-to-computed tomography (CT) times were obtained from the local AHA GWTG-S registry. Clinical data were extracted from prehospital (EMS) records using standardized data collection forms created in REDCap. We abstracted the following variables from the EMS record: transport times, signs and symptoms at the time of presentation, results of stroke screening scale if performed, whether a prehospital notification of suspected stroke was made to the receiving facility, date and time of LKW time if recorded, initial vitals, blood glucose measurements, and the performance of a 12-lead ECG. We defined presentation with classic symptoms that should be detectable by a prehospital stroke screening scale to include unilateral weakness, speech changes, and/or facial droop, as defined by the prehospital stroke screening tools, including the Cincinnati Prehospital Stroke Scale (CPSS) and the Face, Arm, Speech, Time test (Maddali et al., 2018). For this study, we defined guideline-concordant care to include compliance with all eight metrics based on 2019 AHA class 1 recommendations for early management of acute ischemic stroke patients in the prehospital setting (Jauch et al., 2013; Glober et al., 2016; Powers et al., 2019): (1) documentation of stroke scale, (2) prehospital notification of suspected stroke to receiving facility, (3) determination of blood glucose level, (4) completion of a 12-lead ECG, (5) provision of supplemental oxygen if pulse oximetry < 94%; (6) documentation of symptom onset or LKW time, (7) < 2 min from dispatch to being en route, and (8) < 15 min spent on scene (Box 1). While prenotification may represent an intermediated outcome, it was included in the final model given its inclusion in AHA recommendations. Documentation of a formal stroke screening tool was missing in almost 40% of patients with suspected stroke in prior studies. As a surrogate, we also collected information regarding the neurological assessment performed by EMS (Dylla et al., 2022). The data were primarily extracted by H.M.H. and confirmed by L.D. The locations of individual items for extraction were determined *a priori*, and a standard extraction protocol was reviewed before chart extraction. Discrepancies were reviewed by both researchers for consistency.

### Data analysis

To characterize the cohort, we used descriptive statistics, reporting the mean, the standard deviation, the median, the interquartile range for continuous variables, and the frequency and proportion for categorical variables. The COVID-19 pandemic significantly impacted prehospital care in multiple ways, including fewer EMS providers undergoing EMS refresher courses and differences in operating procedures (Velasco et al., 2021; Blek et al., 2022; March et al., 2022). We used a chi-square test to determine the differences in the proportions of encounter compliance with individual recommendations for prehospital care before and during the COVID-19 pandemic (defined as 03/01–12/31/2020). We analyzed compliance with individual metrics in all EMS encounters (including interhospital transfers) and only those involving EMS transport from the scene. We compared the median door-to-CT and the total time from EMS on scene to CT among those compliant and not compliant with individual recommendations using a Mann–Whitney *U* test. Multivariable generalized linear regression modeling identified individual guideline recommendations that were predictive of door-to-CT and EMS on-scene–to–CT times among those patients transported from the scene by EMS overall and before and during the COVID-19 pandemic. Variables included in the model were determined *a priori* and included biological sex, age, stroke severity (National Institutes of Health Stroke Scale, NIHSS), each of the eight individual guideline metrics, documentation of a neurological assessment that was positive for classic stroke symptoms (facial droop, unilateral limb weakness, and/or speech changes), and overall guideline-concordant care (defined as compliance with all seven AHA recommendations for prehospital care of suspected stroke patients). All analyses were performed using SAS v9.4 software (SAS Institute, Cary, NC).

## Results

### Subject demographics and quality of EMS care

Of the initial 621 eligible patients, 4 patients were reported to have arrived by private vehicle (despite having an associated EMS run chart), and 166 were transfers from another hospital. Study sample demographics are included in Table 1. Only 44.8% (*n* = 278) of encounters overall had a documented prehospital stroke scale by EMS, with the majority (65.5%, *n* = 182) being positive (Table 2). However, EMS performed and documented a neurological exam in 96.9% (*n* = 602) of all subjects. Of these assessments, 69.7% (*n* = 433) were positive for one or more classic stroke symptoms (unilateral limb weakness, facial droop, and/or speech changes).

**Table 1:** Subject demographics and stroke characteristics.

**Characteristic**		***N* = 621**
Age in years – mean (*SD*)		66.0 (16.3)
Male Sex – *n* (%)		364 (58.6%)
Race – *n* (%)	White	382 (61.5%)
	Black/African American	139 (22.4%)
	Native Hawaiian/Pacific Islander	2 (0.3%)
	Asian	18 (2.9%)
	American Indian/Alaskan Native	3 (0.5%)
	Unable to Determine	77 (12.4%)
Ethnicity – *n* (%)	Hispanic	80 (12.9%)
NIH Stroke Scale Score upon Admission – mean (*SD*); median (IQR)		8.5 (8.4); 5 (2,14)
Patients Mode of Arrival to the ED^*^	EMS transport from home/scene	452 (72.8%)
	EMS transfer from another hospital	166 (26.7%)
	Private transport from home/scene	4 (0.6%)

n, number subjects; SD, standard deviation; NIH, National Institutes of Health; IQR, interquartile range; ED, emergency department. ^*^Patients transferred from another hospital or arriving by private vehicle were excluded from modeling despite having an associated prehospital run sheet.

**Table 2:** Comparison of door-to-CT times based on compliance with AHA recommendations for prehospital care of suspected stroke patients.

**AHA recommendation**	**Recommendation not met, mean (*SD*) and median (IQR), DTCT**	**Recommendation met, mean (*SD*) and median (IQR), DTCT**
Documented stroke scale	18.7 (20.9); 10 (6, 20)	17.6 (20.2); 9 (5, 18)
Blood glucose obtained^*^	30.0 (26.6); 21 (7,50)	17.0 (19.5); 10 (6,18)
Supplemental oxygen for pulse oximetry < 94%	17.0 (18.8); 10 (6, 19.5)	19.6 (22.3); 10 (5, 20)
12-lead ECG	18.0 (21.1); 8 (6, 18)	18.4 (20.3); 10 (6, 20.5)
Documented LKW or symptom onset time^*^	26.9 (24.7); 16 (7, 44)	12.7 (15.1); 8 (5, 13)
< 2 min from dispatch to en route	15.9 (17.9); 7.5 (4.0, 22.5)	18.3 (20.7); 10 (6, 20)
< 15 min on-scene time	19.9 (21.0); 12 (6, 24)	17.6 (20.4); 9 (5,20)
Prenotification to receiving hospital of suspected stroke^*^	35.3 (24.8); 26.5 (15, 52)	8.8 (8.4); 7 (5, 10)
Fully guideline-concordant care^*^	18.9 (21.0); 10 (6, 21)	7.4 (4.8); 7 (4, 8)
EMS documented classic symptoms (unilateral weakness, facial droop, and/or speech changes)^*^	35.0 (24.3); 30 (15, 52)	12.7 (15.6); 8 (5, 13)

CT, computed tomography; AHA, American Heart Association; DTCT, door-to-CT time (in minutes); ECG, electrocardiogram; LKW, last-known well; IQR, interquartile range; SD, standard deviation. ^*^p < 0.05 on Mann–Whitney U-test.

Among only those patients who were transported by EMS from the scene, EMS compliance with AHA recommendations for prehospital care of suspected stroke patients was highly variable for each recommendation (Supplementary Table S1). Almost all (95.6%, *n* = 432) subjects had an EMS response time from call to dispatch that was < 2 min. The time of symptom onset or LKW was documented by EMS for 58.0% (*n* = 262) of subjects. However, only 48.2% (*n* = 218) of those who received supplemental oxygen did so in a manner as indicated (only for a pulse oximetry < 94% or chronic supplemental oxygen use). Full compliance with all eight recommendations for prehospital care occurred in only 5.1% (*n* = 23) of subjects. More than 60% of encounters were compliant with at least four individual recommendations.

### Comparison of time to CT among encounters compliant and non-compliant with AHA recommendations

Encounters in which a blood glucose was obtained, an LKW time or symptom onset documented, a prenotification of suspected stroke was made, classic symptoms documented, or all AHA guidelines were fully complied with, the median door-to-CT times were significantly shorter compared to those respective non-compliant encounters (Table 2). Similarly, median total times were shorter from EMS arrival on scene to CT when the encounter was compliant for a documented LKW time, an on-scene time < 15 min, a prenotification of a suspected stroke patient, documentation of classic symptoms, or full compliance with all AHA recommendations (Supplementary Table S2).

### Multivariable linear regression to predict door-to-CT time

Multivariable generalized linear regression identified individual recommendations for prehospital care associated with door-to-CT time while controlling for sex, age, and stroke severity (NIHSS score at admission). The final model included 358 (excluding 94 encounters without a door-to-CT time and EMS transfers from another hospital). Among the AHA recommendations, only prehospital notification of suspected stroke to the receiving hospital and documentation of LKW time or symptom onset were associated with shorter door-to-CT times (Table 3). Given the low frequency of a documented stroke scale assessment, we included documentation of a neurological assessment positive for classic stroke symptoms, which was also associated with shorter door-to-CT times, in the model as a surrogate. However, full guideline-concordant care was not associated with shorter door-to-CT time after controlling for confounders. Only an on-scene time of < 15 min and prenotification were observed to have shorter times of EMS arrival to CT when adjusting for age, sex, and initial NIHSS score.

**Table 3:** Multivariable linear regression modeling of door-to-CT times.^*^

**Variable**		**Point estimate, minutes [95% CI]**
Intercept		45.5 [33.8, 57.3]
Predictors		
Patient characteristics	Female vs. male sex	−1.5 [−4.9, 1.9]
	Age (in years)	−0.03 [−0.1, 0.1]
	NIHSS score at admission	0.02 [−7.5, 7.9]
EMS care components	EMS documented classic symptoms	−9.1 [−13.5, −4.7]
	Stroke scale completed by EMS	−0.8 [−4.3, 2.7]
	Prenotification of receiving hospital of suspected stroke	−20.5 [−24.3, −16.6]
	12-lead ECG completed	−2.1 [−5.7, 1.4]
	Blood glucose obtained	−5.0 [−10.9, 0.8]
	Supplemental oxygen provided for pulse oximetry < 94%	3.0 [−0.4, 6.4]
	Documented time of LKW or symptom onset	−5.2 [−8.9, −1.5]
	< 2 min rom call to dispatch	1.8 [−6.3, 10.0]
	< 15 min on-scene time	1.3 [−2.5, 5.2]
	Fully guideline-concordant care	0.2 [−7.5, 7.9]

CI, confidence interval; ECG, electrocardiogram; EMS, emergency medical services; LKW, last-known well; NIHSS, National Institutes of Health Stroke Scale. ^*^Final model including 358 encounters without missing outcome variables.

### Differences in care associated with the COVID-19 pandemic

Among only those transported by EMS from the scene, an analysis of EMS compliance with individual performance metrics before the COVID-19 pandemic (before 03/01/2020) and during the COVID-19 pandemic showed similar proportions of compliance with all individual recommendations except for documentation of LKW time. EMS documented a time of LKW in 61.7% (*n* = 172/279) of encounters before the COVID-19 pandemic and 52.0% (*n* = 90/173) of encounters during the COVID-19 pandemic (*p* = 0.04; Supplementary Table S1). While controlling for sex, age, stroke severity, and the timing of the EMS encounter (during the COVID-19 pandemic compared to before the COVID-19 pandemic) was associated with increased door-to-CT times (Supplementary Table S2).

## Discussion

This study found low rates of full compliance with the AHA guidelines for prehospital care of suspected stroke patients. Encounters with full compliance were observed to have shorter median door-to-CT times, a common measure of in-hospital quality of care in the unadjusted analysis, but this was not observed after adjusting for age, gender, and initial NIHSS score. This study focused on broad compliance with AHA guidelines, rather than individual EMS protocol compliance, to identify components of AHA prehospital stroke care recommendations that were observed to improve door-to-CT times. Median door-to-CT times and total times from EMS arrival on scene to CT were shorter among encounters in which EMS documented LKW time or symptom onset, a prenotification of suspected stroke was made, classic symptoms were documented (regardless of whether a prehospital stroke screening tool was used), or the encounter was fully compliant with recommendations. After controlling for the COVID-19 pandemic, these associations remained. The COVID-19 pandemic was associated with prolonged door-to-CT times overall. The factors identified as being associated with shorter door-to-CT times are key factors in identifying suspected stroke (i.e., documentation of classic stroke symptoms) and help ready in-hospital teams for acute treatments (early identification of LKW time and potential eligibility for acute interventions). They also afford providers with advanced notification that allows them to be present in the ED upon arrival, and they help rule out stroke mimics (such as hypoglycemia).

Door-to-CT time reflects the efficiency of in-hospital care, including patient registration, direct transport to CT, and availability of a multidisciplinary team for ED evaluation. However, these in-hospital protocols can vary based on EMS suspicion of a potential stroke. At the institution studied, prenotification allowed for preregistration of a patient and direct transport to CT. Door-to-CT times have been previously shown to decrease in cases in which EMS suspected a stroke and provided prenotification (Sheppard et al., 2015; Magnusson et al., 2022). Oostema et al. (2014) found that documentation of a CPSS score, an on-scene time ≤ 15 min, documentation of LKW time, hospital prenotification, and highest priority transport were associated with a door-to-CT time of ≤ 25 min. This is supported by the results presented here where shorter door-to-CT times and time between EMS arrival on scene and CT were shorter when EMS was compliant with recommendations for LKW, prenotification, documentation of classic symptoms, or full compliance. That these factors all resulted in shorter times to CT is not surprising. These factors will inherently bias in-hospital teams to not only proceed with a rapid stroke assessment but also provide the key information needed to make these acute diagnostic and treatment decisions.

This study has important implications for the prehospital care of suspected stroke patients. Based on these results, certain questions arise. For instance, is a 12-lead ECG needed in the prehospital setting? It may help detect atrial fibrillation and identify patients at increased risk of stroke, but it may not shorten the time to acute interventions. In contrast, documentation of an LKW time or symptom onset not only shortened the time to CT but is also a critical component of prehospital care – access to bystanders who can provide this information is not always readily available upon ED arrival. Furthermore, this information can help ED personnel decide to call a prehospital stroke alert, thereby expediting time to CT and time to treatment. A previous randomized control trial similarly found that the collection of additional structured data elements by EMS did not necessarily increase the number of patients receiving thrombolysis or shorten the time to thrombolysis (Price et al., 2019). By paring down the recommendations, a focused educational intervention that minimizes prehospital transport times and emphasizes only those components of care that significantly impact the timing and ability to proceed with acute intervention may be more readily accepted by EMS providers and adopted into routine EMS protocols.

This study and existing literature surrounding individual recommendations for obtaining a blood glucose level, incorporating a stroke screening scale or neurological assessment, and prehospital notification of a suspected stroke point to key components for more focused prehospital intervention in stroke. In a 2012–2013 study, compliance with a glucose measurement was the second most frequently performed metric among all strokes (in 86% of encounters) whereas a documented CPSS occurred in 78.5% of encounters (Oostema et al., 2014). More recently, among Michigan EMS encounters for a suspected stroke, measuring the blood glucose level was, again, among the most frequently performed recommendation, with documenting a stroke scale score occurring in just over half of encounters and documenting LKW time in < 30% of encounters (Oostema et al., 2023). Our data show similar rates of obtaining a blood glucose level and prenotifying the receiving facility of a suspected stroke (Oostema et al., 2014).

Among patients with a final diagnosis of stroke, prehospital suspicion of a suspected stroke occurred in 52% of patients, usually in those patients presenting with classic signs and symptoms of a stroke (Andersson et al., 2018). In these cases, prehospital providers were then more likely to obtain a 12-lead ECG and blood glucose. However, both the data presented here and in prior studies showed a low frequency of documenting stroke scale scores by prehospital providers, which can limit the early identification of strokes in the prehospital setting and prehospital notification of a receiving hospital. In our study, a prehospital provider documented a stroke scale among patients with confirmed stroke only 45% of the time. Yet, providers performed a neurological assessment in most patients and documented classic stroke signs and symptoms that are detected in most prehospital stroke screening tools in over two-thirds of patients. However, both these tools and the NIHSS are subject to misdiagnosis, especially in patients presenting with posterior circulation occlusions. Additional research is needed to develop enhanced screening tools that will be more universally adopted by providers. When EMS providers suspect a stroke, most protocols prompt them to complete a prehospital stroke scale assessment. If positive, most protocols will further prompt prenotification of the receiving hospital. The finding that EMS documenting a neurological exam and an LKW time is associated with shorter door-to-CT times suggests that these may be important areas for focused EMS education. These interventions are generally completed en route to the hospital from the scene, so they are unlikely to cause prolonged prehospital transport times while reducing hospital providers' time for acute treatment decisions. Performing a neurological exam to identify common signs of a potential stroke, completing a prehospital stroke scale, and prenotifying the receiving hospital of a suspected stroke may also be three ways to rapidly communicate to an ED a high degree of suspicion of stroke to facilitate rapid evaluation by stroke neurologists.

Prenotification of suspected stroke patients by EMS is part of the protocols that reduce the time to treatment and improve outcomes (Medoro and Cone, 2017). However, in many EMS protocols, prenotification relies on a positive stroke screening scale, as is the case for the regional EMS protocols encompassed by this study. Despite the emphasis placed on prehospital stroke scales and with many scales in use worldwide, no single tool has been identified as clearly superior to others (Zhelev et al., 2019). In the present study, low rates of documentation of any prehospital stroke screening tool suggest an ongoing need for provider education regarding the importance of screening for stroke using the best available tools. The precise reasons for failing to perform a stroke screening scale are unknown. However, focus groups previously identified diversity in stroke presentations as one barrier to recognizing a potential stroke and complying with prehospital care recommendations (Oostema et al., 2019). This is supported by the fact that even among patients with a final diagnosis of stroke, more than a third of the completed stroke scales were reported as negative or inconclusive by the prehospital providers in this study. Instead, our study found that documentation of a neurological assessment that was positive for classic symptoms was associated with shorter door-to-CT times. Relying only on a positive stroke scale to allow prenotification may result in delays in diagnosis and treatment for many patients. Given the low rates of documentation of a stroke scale, documentation of a neurological assessment – which is part of many patient assessments – may serve as an additional tool in stroke screening and hospital prenotification for stroke. The finding that documentation of classic stroke symptoms on a neurological assessment was associated with a shorter door-to-CT time may support an alternative method of prehospital identification of possible patients with a stroke – one that does not rely on providers remembering to perform and document a prehospital stroke screening scale.

Prehospital providers are tasked with stabilizing critically ill patients and identifying those patients that warrant prehospital interventions and notification of receiving facilities for suspected myocardial infarctions and strokes. These complexities of prehospital care necessitate a focus on critical components in care that impact patient outcomes. The expectation that a prehospital provider can fully comply with the numerous AHA recommendations for prehospital care in all possible stroke patients may be unrealistic, given the burden of workload in the back of a moving vehicle. Regardless, provider education emphasizing the importance of a neurological assessment, blood glucose measurement, and documenting LKW not only helps ED providers in acute stroke diagnosis and treatment but also remains relevant to the treatment of numerous other critical illnesses that prehospital and emergency medicine providers will encounter.

## Study limitations

A key limitation of this study is that it only assesses the impact of prehospital care on in-hospital times to CT from a single comprehensive stroke center. Both prehospital and in-hospital providers working with comprehensive stroke center providers may be more familiar with AHA recommendations. In this system, the criteria for prenotification of suspected stroke can vary slightly between EMS agencies, but regional protocols require a positive stroke scale and no evidence of hypoglycemia. Agency-specific protocols allow one set of providers to call a prehospital stroke alert for patients who (1) have an LKW time of < 12 h, (2) have a blood glucose level >60 mg/dL, (3) do not have a new seizure at onset or recent head trauma, and (4) who screen positive on the CPSS. The other agency does not call stroke alerts from the field but, rather, calls the receiving facility to notify of general concerns for a cerebrovascular event based on neurological assessment and an overall clinical picture. Furthermore, the agency that does not use stroke alert criteria also does not universally document stroke scale findings in the patient care report, despite having protocolization of the CPSS. The lack of use of a stroke alert protocol may possibly limit the number of potential strokes called in advance of ED arrival. As such, performing a prehospital stroke scale may be less impactful on door-to-CT times in cases in which providers are allowed to call ahead based on neurological assessment alone. Alternatively, upon a patient's arrival at the ED, providers can quickly initiate a “stroke alert” when supplied with critical information such as a neurological assessment, blood glucose, and LKW. At this hospital, if both prenotification of potential “stroke alert” and stroke-like presentations based on neurological assessment occur, patients may go directly to CT. However, in the absence of a clear use of standardized stroke alert criteria that require the need for more information to be communicated via telephone, some patients may potentially be roomed when, in fact, they meet the parameters for a stroke alert, increasing door-to-CT time. An in-hospital “stroke alert” will prioritize these patients for advanced imaging and result in rapid bedside assessment. Smaller institutions, without robust resources immediately available for all situations, may need to rely more heavily on the results of a prehospital stroke scale and prenotification to make these resources readily available. Additionally, while short door-to-CT times are one component of rapid diagnosis and treatment of acute stroke, many factors contribute to patient outcomes. Door-to-CT is just one metric of in-hospital care that is regularly documented in GWTG-S registries. With variable initial workflows for acute stroke patients (i.e., straight to CT by EMS or a rapid ED evaluation followed by transport to the CT), these times may also reflect some differences in institutional protocols.

## Conclusion

Shorter door-to-CT times at this comprehensive stroke center were observed with EMS documentation of an LKW or time of symptom onset, obtaining a blood glucose level, prenotifying the receiving hospital of suspected stroke, or fully complying with AHA recommendations. Documentation of a neurological assessment that identified classic symptoms of a stroke was also associated with shorter door-to-CT times, while the performance of a stroke screening scale, which was less common, was not associated with shorter times. These findings, combined with low rates of full compliance with AHA guidelines, reaffirm the need for continued education on the important role of the prehospital provider in helping identify strokes and provide timely treatment. Larger studies are needed to confirm these findings and continue to identify best practices for the prehospital care of these critically ill patients.

## Data availability statement

The original contributions presented in the study are included in the article/Supplementary material, further inquiries can be directed to the corresponding author.

## Ethics statement

The studies involving humans were approved by the Colorado Multiple Institutional Review Board. The studies were conducted in accordance with the local legislation and institutional requirements. Written informed consent for participation was not required from the participants or the participants' legal guardians/next of kin in accordance with the national legislation and institutional requirements.

## Author contributions

LD: Conceptualization, Data curation, Formal analysis, Funding acquisition, Methodology, Visualization, Writing—original draft, Writing—review & editing. HH: Data curation, Writing—review & editing. CW: Writing—review & editing, Investigation, Validation. ML: Writing—review & editing. BR: Data curation, Writing—review & editing. KJ: Writing—review & editing, Data curation. HB: Writing—review & editing, Data curation. AM: Writing—review & editing. SP: Conceptualization, Methodology, Writing—review & editing.
