# Supplementary material for: Identification of specific recommendations for prehospital stroke care associated with shorter door-to-CT times – An analysis of Get with the Guidelines-Stroke registry and prehospital data
Source: Front Stroke. 2024 May 30;3:1355889. doi: 10.3389/fstro.2024.1355889 (PMC12288049; doi:10.3389/fstro.2024.1355889)
Supplement: Supplementary file 1 [file Table_1.PDF]

Supplementary Table 1: EMS and In-hospital Care

Characteristic		Overall (Including EMS transports from another hospital) (n=621) N (%)	EMS Transports from Scene (n=452) N (%)
Door to CT time in minutes – median (IQR)*		12.0 (6, 50)	12.0 (6, 50);
Stroke Scale Result**	Positive	182 (65.5%)	111 (58.7%)
	Negative	80 (28.8%)	65 (34.4%)
	Inconclusive	16 (5.8%)	12 (6.9%)
Neurological Exam Documented		602 (96.9%)	441 (97.6%)
Neurological Exam Signs and Symptoms	Unilateral arm weakness	297 (47.8%)	214 (47.4%)
	Unilateral leg weakness	232 (37.4%)	166 (36.8%)
	facial droop	251 (40.4%)	183 (40.5%)
	Speech Changes	300 (48.3%)	216 (47.8%)
	numbness	60 (9.7%)	38 (8.4%)
	Dizziness	57 (9.2%)	47 (10.4%)
	AMS/Confusion	162 (26.1%)	111 (24.6%)
	Headache	56 (9.0%)	38 (8.4%)
	nausea/vomiting	54 (8.7%)	45 (10.0%)
	Vision changes	60 (9.7%)	36 (8.0%)
	Generalized/Bilateral Weakness	60 (9.7%)	48 (10.6%)
	Other (including gait abnormalities, ataxia)	54 (8.7%)	45 (10.0%)
	No Deficits	31 (5.0%)	19 (4.2%)
EMS documented classic symptoms (unilateral weakness, facial droop, and/or speech changes) on neurological exam		433 (69.7%)	312 (69.0%)
EMS compliance with individual AHA recommendations	Documented stroke scale	279 (44.9%)	190 (42.0%)
	Blood glucose obtained	492 (79.1%)	404 (89.4%)
	Supplemental oxygen for pulse oximetry less than 94%	300 (48.2%)	218 (48.2%)
	12-lead ECG	320 (51.5%)	256 (56.6%)
	Documented LKW or symptom onset	363 (58.4.0%)	262 (58.0%)
	<2 minutes from dispatch to enroute	560 (90.2%)	432 (95.6%)
	<15 minutes on-scene time	386 (62.2%)	330 (73.0%)
	Prenotification to Receiving Hospital of Suspected Stroke†	261 (42.0%)	261 (57.7%)
	Fully guideline-concordant care	46 (7.4%)	23 (5.1%)

Abbreviations: AMS – altered mental status; ECG – electrocardiogram; LKW – last known well; IQR – interquartile range

* DTCT missing in 185/621 total encounters; 29 out of 423 EMS transport from scene

** Stroke Scale Result – percentages are out of the number of encounters with a documented stroke scale (see "EMS compliance with individual AHA recommendations"). Results of the stroke scale are reported as the EMS provider reported in the medical record.

† Prenotification to Receiving Hospital of Suspected Stroke – per regional protocols, requires positive stroke screening scale and no hypoglycemia. Missing documentation n=180 overall, with variable excluded from definition of fully guideline-concordant care; missing n=10 of EMS transfer from scene, included in definition of fully guideline-concordant care.

Supplementary Table 2: Comparison of Total time-to-CT times Based on Compliance with AHA Recommendations for Prehospital Care of Suspected Stroke Patients.

AHA Recommendation	Recommendation not Met mean (stdev) and median (IQR) TTCT	Recommendation Met mean (stdev) and median (IQR) TTCT
Documented stroke scale	27.25 (21.50); 20 (14, 31)	27.16 (21.90); 20.50 (12, 35)
Blood glucose obtained	34.5 (28.3); 22.5 (12.5, 54)	26.3 (20.6); 20 (13, 30)
Supplemental oxygen for pulse oximetry less than 94%	26.3 (20.4); 20 (13, 29)	28.3 (23.0); 20 (12, 35)
12-lead ECG	26.2 (22.1); 19.0 (13, 28)	28.0 (21.3); 21 (13, 34)
Documented LKW or symptom onset*	33.1 (25.9); 23 (14, 46.5)	23.0 (16.8); 19 (12.0, 27.5)
<2 minutes from dispatch to enroute	28.7 (19.5); 20 (16, 39)	27.1 (21.8); 20 (13, 32)
<15 minutes on-scene time*	37.8 (21.6); 29.5 (23.0, 44.0)	23.1 (20.3); 16 (11, 25)
Prenotification to Receiving Hospital of Suspected Stroke†*	39.1 (26.9); 32 (18, 60)	19.1 (11.6); 17 (12, 23)
Fully guideline-concordant care*	28.0 (21.9); 21 (13, 33)	13.3 (5.7); 12 (8, 17)
EMS documented classic symptoms (unilateral weakness, facial droop, and/or speech changes)*	37.05 (26.7); 30 (16, 58)	22.95 (17.44); 18 (12, 27)

Abbreviations: AMS – altered mental status; TTCT – total time to CT time (time for EMS arrival onscene to CT in minutes); ECG – electrocardiogram; LKW – last known well; IQR – interquartile range; stdev (standard deviation)

*p<0.05 Mann-Whitney U test

Supplementary Table 3: Multivariable Linear Regression Modeling of Total Time to CT Times*

**Variable**		**Point Estimate, Minutes (95% CI)**
Intercept		50.6 (37.9, 63.3)
Predictors		
Patient Characteristics	Female vs male sex	-1.4 (-5.1, 2.3)
	Age (in years)	-0.04 (-0.2, 0.1)
	NIHSS score at admission	0.13 (-0.1, 0.36)
EMS Care Components	EMS documented classic symptoms	-4.6 (-9.2, 0.1)
	Stroke scale completed by EMS	0.6 (-3.2, 4.5)
	Prenotification of receiving hospital of suspected stroke	-14.8 (-19.0, -10.6)
	12-lead ECG completed	-0.3 (-4.1, 3.61)
	Blood glucose obtained	-2.3 (-8.4, 3.8)
	Supplemental oxygen provided for pulse oximetry less than 94%	3.9 (0.2, 7.7)
	Documented time of last known well or symptom onset	-2.4 (-6.4, 1.64)
	<2 minutes rom call to dispatch	0.6 (-8.4, 9.4)
	<15 minutes on-scene time	-10.9 (-15.1, -6.8)
	Fully guideline-concordant care	-5.0 (-18.8, 3.75)

* Final model including 358 encounters without missing outcome variables.
Abbreviation: CI – Confidence Interval; Total time to CT – time EMS onscene to CT

Supplementary Table 4: EMS Compliance with Individual AHA Recommendations Before and During the Covid-19 Pandemic

**Recommendation**	**Before Covid-19 Pandemic** (n=279) N (%)	**During Covid-19 Pandemic** (n=173) N (%)
Documented stroke scale	116 (41.6%)	74 (42.8%)
Prenotification of receiving hospital of suspected stroke	154 (55.6%)	108 (64.7%)
Blood glucose obtained	245 (87.8%)	159 (91.9%)
Supplemental oxygen for pulse oximetry less than 94%	130 (46.6%)	88 (50.9%)
12-lead ECG	154 (55.2%)	102 (59.0%)
Documented LKW or symptom onset*	172 (61.7%)	90 (52.0%)
<2 minutes from dispatch to enroute	265 (95.0%)	167 (96.5%)
<15 minutes on-scene time	201 (72.0%)	129 (74.6%)
Fully guideline-concordant care	11 (3.9%)	12 (6.9%0

*chi-square p<0.05

Supplementary Table 5: Multivariable Linear Regression Modeling of Door-to-CT Times including Covid-19 pandemic as covariate*

**Variable**		**Point Estimate, Minutes (95% CI)**
Intercept		45.6 (34.0, 57.2)
Predictors		
Patient Characteristics	Female vs male sex	-1.1 (-4.5, 2.3)
	Age (in years)	-0.05 (-0.1, 0.1)
	NIHSS score at admission	0.1 (-0.3, 0.2)
	Encounter during vs before Covid-19 pandemic	4.7 (1.3, 8.0)
EMS Care Components	EMS documented classic symptoms	-9.1 (-13. 4, -4.7)
	Stroke scale completed by EMS	-0.9 (-4.3, 2.6)
	Prehospital notification of suspected stroke to receiving facility	-20.9 (-24.8, -17.1)
	12-lead ECG completed	-2.1 (-5.6, 1.5)
	Blood glucose obtained	-5.7 (-11.5, 0.2)
	Supplemental oxygen provided for pulse oximetry less than 94%	2.8 (-0.6, 6.2)
	Documented time of last known well or symptom onset	-4.6 (-8.3, -1.0)
	<2 minutes rom call to dispatch	1.6 (-6.4, 9.7)
	<15 minutes on-scene time	1.4 (-2.4. 5.3)
	Fully guideline-concordant care	-0.02 (-0.2, 0.2)

*Final model including 352 encounters without missing explanatory or outcome variables.
